# Supplementary material for: Delta Radiomics and Tumor Size: A New Predictive Radiomics Model for Chemotherapy Response in Liver Metastases from Breast and Colorectal Cancer
Source: Tomography. 2025 Feb 20;11(3):20. doi: 10.3390/tomography11030020 (PMC11945686; doi:10.3390/tomography11030020)
Supplement: Supplementary file 1 [file tomography-11-00020-s001.zip › tomography-3417339-supplementary.pdf]

Table S1. Relevant radiomics articles on liver metastases from colorectal and breast cancer predicting tumor response after systemic treatment.

AUTHORS	DOI	YEAR	TIME POINTS	PATIENTS (LESIONS)	STUDY DESIGN	MODALITY	MAIN INTERVENTION
COLORECTAL CANCER							
Dohan et al. [37]	doi.org/10.1136/gutjnl-2018-316407	2018	Baseline, 2 months	270	Retrospective, multicenter	CT	NAC/ bevacizumab
Ravanelli et al. [38]	doi.org/10.1007/s11547-019-01046-4	2019	Baseline, 3 months	43 (135)	Retrospective, single center	CT	NAC/ bevacizumab
Andersen et al. [39]	doi.org/10.1177/0284185118817940	2019	Baseline, 1, 2, 4 months	27 (27)	Retrospective, multicenter	DCE-CT CT	Regorafenib
Wei et al. [40]	doi.org/10.1002/mp.14563	2020	baseline, 8-12 weeks	192 (192)	Retrospective, single center	CT	NAC
Giannini et al. [41]	http://doi.org/10.1109/EMBC44109.2020.9176627	2020	baseline, 12 weeks	24 (84)	Retrospective, single center	CT	NAC
Dercle et al. [42]	doi.org/10.1093/jnci/djaa017	2020	baseline, 8 weeks	538	Retrospective, single center	CT	NAC/ bevacizumab
Lu et al. [19]	doi.org/10.1038/s41467-021-26990-6	2021	baseline, 8 weeks	2190	Retrospective, multicenter	CT	NAC/ aflibercept
Ma et al. [43]	doi.org/10.3748/wjg.v27.i38.6465	2021	baseline, 3 months	102 (223)	Retrospective, single center	MRI	NAC
Giannini et al. [14]	doi.org/10.3390/cancers14010241	2022	baseline, 8 weeks	57 (242)	Retrospective, two center	CT	NAC
Ye et al. [18]	doi.org/10.3389/fonc.2022.843991	2022	baseline, 3 months	139 (397)	Retrospective, single center	CT	NAC
BREAST CANCER							
Fan et al. [44]	doi.org/10.3389/fmolb.2021.622219	2021	baseline, 2 cycles	114 (114)	Retrospective, single center	DCE-MRI	NAC
Guo et al. [45]	doi.org/10.3390/cancers14143515	2022	baseline, 6-8 cycles	140 (140)	Retrospective, single center	DCE-MRI	NAC/ anti-HER2 drugs
Yang et al. [46]	doi.org/10.3389/fonc.2022.748008	2022	baseline, 2 cycles	217 (217)	Retrospective, single center	US	NAC
Miao et al. [47]	doi.org/10.3389/fonc.2022.852809	2022	baseline	83 (83)	Retrospective, single center	DCE-CT	Anti-HER2

Table S2. The distribution of therapy regimens within each cancer group.

Breast cancer		Colon cancer	
Capecitabine	11% (6)	FOLFOX + Bevacizumab	41% (15)
Capecitabine / Paclitaxel	4% (2)	FOLFOX	22% (8)
Capecitabine / Pembrolizumab	4% (2)	FOLFOX + Cetuximab	5% (2)
Carboplatin / Paclitaxel	2% (1)	FOLFOX + Trastuzumab	8% (3)
Eribulin / Doxorubicin	2% (1)	Floxuridine	3% (1)
Fulvestrant / Alpelisib	4% (2)	Capecitabine	3% (1)
Fulvestrant / BET inhibitor	2% (1)	Capecitabine + Oxaliplatin	5% (2)
Fulvestrant / Denosumab	2% (1)	Irinotecan	3% (1)
Ribociclib / Fulvestrant	2% (1)	Other	11% (5)
Ribociclib / Leuprorelin	2% (1)		
Paclitaxel	4% (2)		
Paclitaxel / Pertuzumab / Trastuzumab	2% (1)		
Paclitaxel / Trastuzumab / Doxorubicin / Cyclophosphamide	2% (1)		
Palbociclib / Anastrozole	2% (1)		
Palbociclib / Capecitabine / Fulvestrant	2% (1)		
Palbociclib / Exemestane	2% (1)		
Palbociclib / Fulvestrant	18% (10)		
Palbociclib / Letrazole	21% (12)		
Palbociclib / Letrozole / Leuprorelin	2% (1)		
Pertuzumab / Trastuzumab	4% (2)		
Pertuzumab / Trastuzumab / Everolimus / Vinorelbine	2% (1)		
Pertuzumab / Trastuzumab	2% (1)		
Other	5% (3)		

Table S3. The scanner characteristics.

	**SOMATOM Sensation 64**	**SOMATOM Definition**	**SOMATOM Definition AS**	**SOMATOM X.cite**	**SOMATOM Drive**	**SOMATOM Force**
Type	Single Source	Single Source	Single Source	Dual Source	Dual Source	Dual Source
X-ray tube	Straton	Straton MX-P	Straton MX-P	Vectron	Straton MX	Vectron
Detector	Ultra Fast Ceramic	Ultra Fast Ceramic	Ultra Fast Ceramic	Stellar Infinity	Stellar Infinity	Stellar Infinity
Reconstruction method	SAFIRE	SAFIRE	SAFIRE	ADMIRE	ADMIRE	ADMIRE
Number of acquired slices	64	128	64	128	256	384
In-plane spatial resolution, mm	0.24	0.3	0.33	0.3	0.3	0.24
Rotation time, sec	0.33	0.28	0.33	0.3	0.25	0.25
In-plane temporal resolution, ms	160	142	83	150	75	66
Generator power, kW	80	100	100	105	100	120
Max. scan speed, mm/s	87	87	200	217	458	737
Max. mA	580	800	800	1,200	750	1300
Tube voltage, kV	80,100, 120, 140	70, 80, 100, 120, 140	80, 100, 120, 140	70, 80, 90	70 – 140	70 – 150
Focal spot size, mm	0.6 x 0.6	0.7 x 0.7	0.7 x 0.7	0.6 x 0.7	0.7 x 0.7	0.4 x 0.5

Table S4. List of radiomics features extracted with LIFEx software.

CONVENTIONAL_HUmin
CONVENTIONAL_HUmean
CONVENTIONAL_HUstd
CONVENTIONAL_HUmax
CONVENTIONAL_HUQ1
CONVENTIONAL_HUQ2
CONVENTIONAL_HUQ3
CONVENTIONAL_HUSkewness
CONVENTIONAL_HUKurtosis
CONVENTIONAL_HUExcessKurtosis
DISCRETIZED_HUmin
DISCRETIZED_HUmean
DISCRETIZED_HUstd
DISCRETIZED_HUmax
DISCRETIZED_HUQ1
DISCRETIZED_HUQ2
DISCRETIZED_HUQ3
DISCRETIZED_HUSkewness
DISCRETIZED_HUKurtosis
DISCRETIZED_HUExcessKurtosis
DISCRETIZED_HISTO_Skewness
DISCRETIZED_HISTO_Kurtosis
DISCRETIZED_HISTO_ExcessKurtosis
DISCRETIZED_HISTO_Entropy_log10
DISCRETIZED_HISTO_Entropy_log2
DISCRETIZED_HISTO_Energy[=Uniformity]
SHAPE_Volume(mL)
SHAPE_Sphericity[onlyFor3DROI])
SHAPE_Surface(mm2)[onlyFor3DROI]
SHAPE_Compacity[onlyFor3DROI]
GLCM_Homogeneity[=InverseDifference]
GLCM_Energy[=AngularSecondMoment]
GLCM_Contrast[=Variance]
GLCM_Correlation
GLCM_Entropy_log10
GLCM_Entropy_log2[=JointEntropy]
GLCM_Dissimilarity
GLRLM_SRE
GLRLM_LRE
GLRLM_LGRE
GLRLM_HGRE
GLRLM_SRLGE
GLRLM_SRHGE
GLRLM_LRLGE
GLRLM_LRHGE
GLRLM_GLNU
GLRLM_RLNU
GLRLM_RP
NGLDM_Coarseness

NGLDM_Contrast
NGLDM_Busyness
GLZLM_SZE
GLZLM_LZE
GLZLM_LGZE
GLZLM_HGZE
GLZLM_SZLGE
GLZLM_SZHGE
GLZLM_LZLGE
GLZLM_LZHGE
GLZLM_GLNU
GLZLM_ZLNU
GLZLM_ZP

Table S5. LogitBoost model performance for pretreatment radiomics, delta radiomics, and functional radiomics response assessment models computed for patients with CRC and BC liver metastasis.

			Sensitivity	Specificity	PPV	NPV	Balanced Accuracy
Colorectal Cancer	Pretreatment Radiomics	PD	0.32 ± 0.32	0.99 ± 0.02	0.54 ± 0.48	0.92 ± 0.13	0.65 ± 0.16
		SD	0.91 ± 0.06	0.27 ± 0.17	0.80 ± 0.05	0.49 ± 0.27	0.59 ± 0.09
		PR	0.24 ± 0.20	0.92 ± 0.06	0.35 ± 0.31	0.87 ± 0.04	0.58 ± 0.10
	Delta Radiomics	PD	0.81 ± 0.24	0.98 ± 0.03	0.85 ± 0.20	0.98 ± 0.02	0.89 ± 0.12
		SD	0.91 ± 0.06	0.58 ± 0.14	0.87 ± 0.04	0.70 ± 0.17	0.74 ± 0.08
		PR	0.43 ± 0.18	0.94 ± 0.04	0.61 ± 0.24	0.90 ± 0.03	0.69 ± 0.09
	Baseline-referenced Delta Radiomics	PD	0.71 ± 0.24	0.96 ± 0.04	0.70 ± 0.23	0.97 ± 0.02	0.83 ± 0.12
		SD	0.87 ± 0.07	0.62 ± 0.15	0.88 ± 0.04	0.63 ± 0.14	0.75 ± 0.08
		PR	0.58 ± 0.24	0.93 ± 0.05	0.62 ± 0.20	0.93 ± 0.04	0.75 ± 0.12
Breast Cancer	Pretreatment Radiomics	PD	0.47 ± 0.17	0.84 ± 0.09	0.56 ± 0.16	0.80 ± 0.06	0.65 ± 0.08
		SD	0.71 ± 0.13	0.45 ± 0.14	0.61 ± 0.08	0.57 ± 0.14	0.58 ± 0.09
		PR	0.27 ± 0.21	0.92 ± 0.06	0.41 ± 0.34	0.87 ± 0.04	0.59 ± 0.11
	Delta Radiomics	PD	0.87 ± 0.11	0.95 ± 0.05	0.89 ± 0.11	0.95 ± 0.04	0.91 ± 0.06
		SD	0.87 ± 0.09	0.81 ± 0.10	0.86 ± 0.07	0.85 ± 0.09	0.84 ± 0.06
		PR	0.68 ± 0.24	0.96 ± 0.04	0.77 ± 0.20	0.95 ± 0.03	0.82 ± 0.12
	Baseline-referenced Delta Radiomics	PD	0.93 ± 0.08	0.96 ± 0.04	0.92 ± 0.08	0.97 ± 0.03	0.95 ± 0.04
		SD	0.87 ± 0.08	0.83 ± 0.09	0.87 ± 0.06	0.85 ± 0.08	0.85 ± 0.05
		PR	0.63 ± 0.21	0.95 ± 0.04	0.73 ± 0.19	0.94 ± 0.04	0.79 ± 0.10

Table S6. pcaNNet model (Neural Networks with a Principal Component step) performance for pretreatment radiomics, delta radiomics, and functional radiomics response assessment models computed for patients with CRC and BC liver metastasis.

			Sensitivity	Specificity	PPV	NPV	Balanced Accuracy
Colorectal Cancer	Pretreatment Radiomics	PD	0.08 ± 0.17	0.98 ± 0.05	0.10 ± 0.23	0.91 ± 0.01	0.53 ± 0.08
		SD	0.93 ± 0.11	0.11 ± 0.18	0.76 ± 0.03	0.13 ± 0.20	0.52 ± 0.06
		PR	0.10 ± 0.17	0.95 ± 0.08	0.10 ± 0.18	0.85 ± 0.02	0.52 ± 0.06
	Delta Radiomics	PD	0.28 ± 0.32	0.97 ± 0.05	0.33 ± 0.37	0.93 ± 0.03	0.62 ± 0.15
		SD	0.88 ± 0.12	0.30 ± 0.27	0.80 ± 0.05	0.34 ± 0.29	0.59 ± 0.10
		PR	0.28 ± 0.28	0.92 ± 0.08	0.29 ± 0.29	0.88 ± 0.04	0.60 ± 0.11
	Baseline-referenced Delta Radiomics	PD	0.50 ± 0.36	0.98 ± 0.04	0.59 ± 0.40	0.95 ± 0.03	0.74 ± 0.18
		SD	0.92 ± 0.07	0.61 ± 0.25	0.88 ± 0.07	0.70 ± 0.25	0.77 ± 0.12
		PR	0.64 ± 0.30	0.95 ± 0.05	0.68 ± 0.30	0.94 ± 0.05	0.80 ± 0.14
Breast Cancer	Pretreatment Radiomics	PD	0.27 ± 0.21	0.85 ± 0.14	0.35 ± 0.28	0.74 ± 0.05	0.56 ± 0.08
		SD	0.75 ± 0.20	0.29 ± 0.22	0.57 ± 0.06	0.63 ± 0.26	0.52 ± 0.07
		PR	0.13 ± 0.20	0.93 ± 0.09	0.13 ± 0.19	0.85 ± 0.03	0.53 ± 0.08
	Delta Radiomics	PD	0.81 ± 0.15	0.91 ± 0.07	0.81 ± 0.14	0.93 ± 0.05	0.86 ± 0.07
		SD	0.77 ± 0.13	0.70 ± 0.16	0.77 ± 0.09	0.74 ± 0.11	0.74 ± 0.07
		PR	0.46 ± 0.31	0.92 ± 0.07	0.44 ± 0.28	0.90 ± 0.05	0.69 ± 0.14
	Baseline-referenced Delta Radiomics	PD	0.84 ± 0.16	0.93 ± 0.05	0.86 ± 0.10	0.94 ± 0.05	0.89 ± 0.08
		SD	0.80 ± 0.12	0.69 ± 0.14	0.77 ± 0.07	0.76 ± 0.11	0.75 ± 0.07
		PR	0.40 ± 0.30	0.92 ± 0.07	0.52 ± 0.21	0.89 ± 0.05	0.66 ± 0.13

Table S7. svmRadial model (Support Vector Machines with Radial Basis Function Kernel) performance for pretreatment radiomics, delta radiomics, and functional radiomics response assessment models computed for patients with CRC and BC liver metastasis.

			**Sensitivity**	**Specificity**	**PPV**	**NPV**	**Balanced Accuracy**
Colorectal Cancer	Pretreatment Radiomics	PD	0.17 ± 0.24	0.99 ± 0.03	0.28 ± 0.40	0.92 ± 0.02	0.58 ± 0.11
		SD	0.94 ± 0.09	0.14 ± 0.18	0.77 ± 0.03	0.27 ± 0.33	0.54 ± 0.07
		PR	0.12 ± 0.19	0.96 ± 0.06	0.19 ± 0.29	0.86 ± 0.03	0.54 ± 0.08
	Delta Radiomics	PD	0.33 ± 0.33	0.98 ± 0.03	0.48 ± 0.43	0.97 ± 0.03	0.66 ± 0.16
		SD	0.92 ± 0.08	0.38 ± 0.26	0.82 ± 0.06	0.90 ± 0.03	0.65 ± 0.11
		PR	0.41 ± 0.29	0.94 ± 0.06	0.50 ± 0.33	0.63 ± 0.16	0.67 ± 0.13
	Baseline-referenced Delta Radiomics	PD	0.50 ± 0.38	0.98 ± 0.03	0.63 ± 0.41	0.94 ± 0.03	0.74 ± 0.19
		SD	0.94 ± 0.06	0.59 ± 0.26	0.88 ± 0.07	0.55 ± 0.31	0.77 ± 0.12
		PR	0.63 ± 0.27	0.96 ± 0.04	0.74 ± 0.26	0.90 ± 0.04	0.79 ± 0.13
Breast Cancer	Pretreatment Radiomics	PD	0.19 ± 0.20	0.90 ± 0.12	0.29 ± 0.30	0.73 ± 0.04	0.54 ± 0.07
		SD	0.83 ± 0.19	0.22 ± 0.23	0.57 ± 0.05	0.73 ± 0.26	0.53 ± 0.05
		PR	0.13 ± 0.20	0.95 ± 0.07	0.15 ± 0.24	0.85 ± 0.03	0.54 ± 0.08
	Delta Radiomics	PD	0.73 ± 0.21	0.94 ± 0.06	0.83 ± 0.18	0.90 ± 0.07	0.83 ± 0.10
		SD	0.85 ± 0.10	0.66 ± 0.20	0.76 ± 0.09	0.81 ± 0.11	0.75 ± 0.08
		PR	0.49 ± 0.28	0.95 ± 0.05	0.60 ± 0.30	0.91 ± 0.05	0.72 ± 0.13
	Baseline-referenced Delta Radiomics	PD	0.76 ± 0.20	0.96 ± 0.04	0.90 ± 0.10	0.91 ± 0.06	0.86 ± 0.10
		SD	0.87 ± 0.10	0.65 ± 0.18	0.77 ± 0.08	0.82 ± 0.11	0.76 ± 0.08
		PR	0.46 ± 0.28	0.94 ± 0.05	0.65 ± 0.22	0.90 ± 0.04	0.70 ± 0.13
